# Is YouTube a sufficient source of information on Sarcoidosis?

**DOI:** 10.1186/s12931-024-02956-2

**Published:** 2024-09-09

**Authors:** Katharina Buschulte, Sarah El-Hadi, Philipp Höger, Claudia Ganter, Marlies Wijsenbeek, Nicolas Kahn, Katharina Kriegsmann, Gillian C. Goobie, Christopher J. Ryerson, Markus Polke, Franziska Trudzinski, Michael Kreuter

**Affiliations:** 1 Center for Interstitial and Rare Lung Diseases, Pneumology and Respiratory Critical Care Medicine, University of Heidelberg, German Center for Lung Research (DZL), Thoraxklinik, Heidelberg, Germany; 2 Department of Hematology, Oncology and Rheumatology, Innere Medizin V, Heidelberg University Hospital, Heidelberg, Germany; 3 Center for Interstitial Lung Diseases and Sarcoidosis, Department of Respiratory Medicine, Erasmus MC-University Medical, Center Rotterdam, Rotterdam, the Netherlands; 4 Laborarztpraxis Rhein-Main MVZ GbR, Limbach Gruppe SE, Frankfurt am Main, Germany; 5 Division of Respiratory Medicine, Department of Medicine, University of British Columbia and St. Paul’s Hospital, Vancouver, BC Canada; 6 Centre for Heart Lung Innovation, St. Paul’s Hospital, University of British Columbia, Vancouver, BC Canada; 7 Division of Pulmonary, Allergy, Critical Care and Sleep Medicine, School of Medicine, University of Pittsburgh, Pittsburgh, PA USA; 8 Mainz Center for Pulmonary Medicine, Department of Pneumology, ZfT, Mainz University Medical Center and Department of Pulmonary, Critical Care & Sleep Medicine, Marienhaus Clinic Mainz, Mainz, Germany

**Keywords:** Sarcoidosis, Information, YouTube, Videos, Quality, Content

## Abstract

**Background:**

The internet is a common source of health information for patients and caregivers. To date, content and information quality of YouTube videos on sarcoidosis has not been studied. The aim of our study was to investigate the content and quality of information on sarcoidosis provided by YouTube videos.

**Methods:**

Of the first 200 results under the search term “sarcoidosis,” all English-language videos with content directed at patients were included. Two independent investigators assessed the content of the videos based on 25 predefined key features (content score with 0–25 points), as well as reliability and quality (HONCode score with 0–8 points, DISCERN score with 1–5 points). Misinformation contained in the videos was described qualitatively.

**Results:**

The majority of the 85 included videos were from an academic or governmental source (*n* = 63, 74%), and median time since upload was 33 months (IQR 10–55). Median video duration was 8 min (IQR 3–13) and had a median of 2,044 views (IQR 504 − 13,203). Quality assessment suggested partially sufficient information: mean HONCode score was 4.4 (SD 0.9) with 91% of videos having a medium quality HONCode evaluation. Mean DISCERN score was 2.3 (SD 0.5). Video content was generally poor with a mean of 10.5 points (SD 0.6). Frequently absent key features included information on the course of disease (6%), presence of substantial geographical variation (7%), and importance of screening for extrapulmonary manifestations (11%). HONCode scores were higher in videos from academic or governmental sources (*p* = 0.003), particularly regarding “transparency of sponsorship” (*p* < 0.001). DISCERN and content scores did not differ by video category.

**Conclusions:**

Most YouTube videos present incomplete information reflected in a poor content score, especially regarding screening for extrapulmonary manifestations. Quality was partially sufficient with higher scores in videos from academic or governmental sources, but often missing references and citing specific evidence. Improving patient access to trustworthy and up to date information is needed.

**Supplementary Information:**

The online version contains supplementary material available at 10.1186/s12931-024-02956-2.

## Background

Sarcoidosis is a rare and complex disease with heterogeneous organ manifestations and variable prognosis [1]. The diagnosis is made based on a combination of symptoms, radiology, and histology, and exclusion of differential diagnoses [2]. Course of disease varies, including both an acute course with spontaneous remission and a chronic course with a significant impairment of quality of life and organ function [1, 3]. In this regard, it was shown in a cohort of 81 patients that lymphocyte subpopulation values at the time of diagnosis do not correlate with the severity of the disease [4]. Depending on the organ affected, a variety of symptoms can occur, including dyspnea and cough for pulmonary sarcoidosis as well as more diffuse symptoms such as fatigue, arthralgia, and body pain [5]. Indication for treatment is based on risk for organ damage, symptom burden, and risk for side effects caused by medication [5]. Treatment decisions are often challenging given the lack of high-quality evidence supporting most treatment options [6]. In addition, progression despite treatment is possible and occurs in 20–30% of patients, mainly due to advanced pulmonary fibrotic sarcoidosis and cardiac involvement [7]. Recently, a potential influence of high density lipoprotein cholesterol (HDLC) and total cholesterol (TC) on the risk of sarcoidosis was found, so that these could possibly be used as predictors [8].

The internet is a common source of health information, especially in rare diseases [9]. A German questionnaire-based study showed 94% of patients with sarcoidosis use the internet as a source of information on their disease [10]. However, Goobie et al. reported for idiopathic pulmonary fibrosis (IPF), a rare interstitial lung disease, that YouTube videos frequently provide incomplete and inaccurate information [11]. Wilkens et al. analysed YouTube videos on lymphangioleiomyomatosis (LAM), another very rare cystic lung disease, and found mostly poor video quality and reliability [12]. Similar to IPF and LAM, obtaining complete and correct information is challenging in sarcoidosis due to the complexity and variability of the disease [13]. An analysis of internet resources on sarcoidosis revealed relevant deficiencies in terms of content, but especially on quality of information [14]. These deficiencies are especially critical given that patients with sarcoidosis identify as one of their most important needs as being well-informed about their disease [9].

The aim of our present study was to systematically investigate the content and quality of information on sarcoidosis provided by YouTube videos based on different validated instruments. This has not been studied to date. Our objective was to identify deficits and knowledge gaps that future online information about sarcoidosis can seek to address.

## Methods

The design of our study was adapted from methods developed in prior work [11, 12], and was approved by the Ethics Committee of the Medical Faculty of the University of Heidelberg, Germany (S-435/2021).

### Search strategy and video selection

The initial search was performed on October 4, 2022. Firstly, all cookies and web browsers’ history were deleted. Consequently, the U.S. version of YouTube (youtube.com) was searched for the term “sarcoidosis” and the first 200 hits were saved. These videos were screened systematically for inclusion criteria by one author (SEH). Videos were included if presenting content related to definition, symptoms, risk factors, evaluation, management, or outcome of sarcoidosis. Videos were excluded if they were duplicates, not in English-language, not relevant to sarcoidosis, required registration or enrolment fees to view, or were clearly directed to a non-patient audience.

### Data extraction and video evaluation

Baseline information about the included videos were captured: uniform resource locator (URL), video title, search rank, date of upload, days since upload, host continent and country, video duration, number of views, likes, dislikes, and comments. Viewing rate was defined as number of views divided by number of days since upload multiplied by 100% [15]. Engagement rate was calculated using the sum of the numbers of likes, dislikes, and comments divided by the number of views multiplied by 100% [11]. Videos were assigned to one of five source categories academic/governmental organisations, news/media, industry/for-profit, independent medical professional users, and non-medical users (e.g. blogs, commentaries etc.).

Two experienced investigators (SEH and KB) independently evaluated content and quality of included videos based on both visual and auditory information. Content was assessed by 25 key features, which were defined based on current guidelines together with experts in this field (MW, NK, MK) [2, 5, 16]. Content categories, as defined by a prior study evaluating the quality of internet resources on sarcoidosis-related information [14], included definition, symptoms, risk factors, evaluation, management, and outcome. Key features were scored as either fully addressed (1 point), partially addressed (0.5 points) or not addressed (0 points), and a summed content score was assigned for each video. All incorrect or misleading facts were qualitatively collected.

Quality analysis was performed using the Health on the Net Code (HONCode), which is provided by an independent organisation for quality assessment of health information. HONCode score is based on eight principles rated with scores from 0 for non-fulfilled to 1 for fulfilled (supplement S1) [17]. Subsequently, the HONCode score for each video was evaluated as low (0–2 points), medium (3–5 points), or high (6–8 points) quality. DISCERN score was determined for each video, which is validated for the quality assessment of user-focused health information on treatment [18]. The DISCERN instrument contains 16 questions, including 8 on reliability, 7 on specific details regarding treatment choices, and 1 on overall quality. Scoring for individual DISCERN items range from 1 (quality criterion has not been fulfilled) to 5 (completely fulfilled), with total DISCERN score ranging from 16 to 80 (supplement S2) [18].

Disagreements between raters of each video were defined as a difference of > 1 point for each DISCERN items and > 0.5 points for key features. In these cases, a re-evaluation of the videos was performed until agreement was reached.

### Statistical analysis

Statistical analysis was performed in a descriptive manner. General information on the videos was provided as absolute numbers and percentages; medians/ interquartile range (IQR) were used for non-normally distributed variables and means/ standard deviation (SD) for normally distributed variables. In order to compare videos from different source categories, news/media, industry/for-profit organisation, and non-medical users (blogs, commentary) were grouped into an “other” category due to low video numbers in each category. For between-group differences of videos by video category Kruskal-Wallis test, Fisher test, and ANOVA were used as appropriate. This was performed for general video information, and all items of content score and both quality scores as well as the summed scores. In addition, HONCode score and DISCERN score of each video grouped by video category were illustrated in a heat map. Multivariable linear regression analysis was used to evaluate the relationship between independent variables video ranking (video search rank order) and viewing rate in the context of the independent variables HONCode score, DISCERN score, and content score. Unadjusted analyses were performed for the associations between HONCode score, DISCERN score, content score, viewing rate, and video ranking. Statistical significance was defined as a two-sided at p value < 0.05 for all analyses. Excel and RStudio 2022.12.0 were used for all analyses.

## Results

### Video selection

**Fig. 1:**
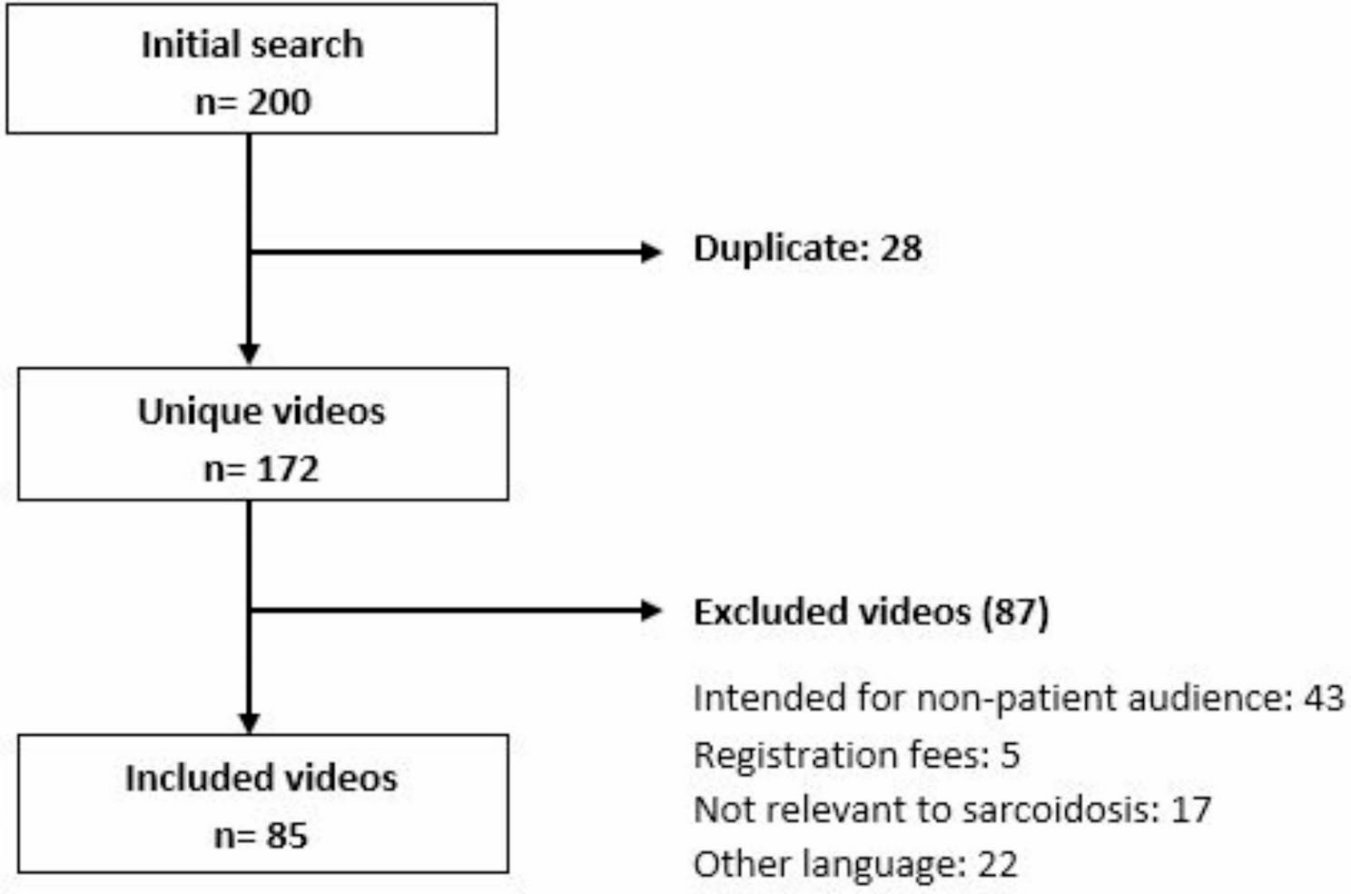
Search results and selection for English videos

The first 200 YouTube videos on the search term “sarcoidosis” were assessed with 85 videos included in the final analysis (Fig. 1, supplement S3).

### Video characteristics

Characteristics of unique videos are listed in Table 1. Most videos were from an academic or governmental source (*n* = 63, 74%), followed by 11% from independent medical professional users (*n* = 9). The hosts were mainly located in the US (*n* = 64, 75%) and UK (*n* = 12, 14%). Median time since upload was 33 months (10–55). Median video duration was 8 min (3–13), and the videos had 2044 views (504–13203). A median of 39 likes (10–172), 0 dislikes (0–3), and 2 comments (0–17) were noted. Median viewing rate was calculated as 266 (108–941), and median engagement rate of 2 (1–3; Table 1).

**Table 1:** Characterization of unique videos – general information, quality and content of medical information on sarcoidosis

Overall unique videos, *n* (%)			85 (100)
General information	Video category, n (%)	Academic/ governmental	63 (74)
		News/media	5 (6)
		Industry/for-profit organisation	2 (2)
		independent medical professional users	9 (11)
		non-medical users (blogs, commentary)	6 (7)
	Host continent, n (%)	Europe	12 (14)
		North America	66 (78)
		South America	0 (0)
		Asia	3 (3.5)
		Australia	1 (1)
		Africa	0 (0)
		Antartica	0 (0)
		Not given	3 (3.5)
	Time since upload in months	Median [IQR]	33 [10, 55]
	Video duration in minutes	Median [IQR]	8 [3, 13]
	Views	Median [IQR]	2044 [504, 13203]
	Likes	Median [IQR]	39 [10, 172]
	Dislikes	Median [IQR]	0 [0, 3]
	Comments	Median [IQR]	2 [0, 17]
	Viewing rate	Median [IQR]	266 [108, 941]
	Engagement rate	Median [IQR]	2 [1, 3]
General quality	HONCode	HONCode score, mean (SD)	4.4 (0.9)
		High, n (%)	8 (9)
		Medium, n (%)	77 (91)
		Low, n (%)	0 (0)
	DISCERN score	Mean (SD)	2.3 (0.5)
General content	Content score	Mean (SD)	10.5 (0.6)
	Websites with incorrect or misleading facts	n (%)	8 (9)

Data is shown as n (%), median (IQR) or mean (SD). HON, Health on the Net; IQR, interquartile range; SD, standard deviation

Videos from academic/governmental sources and independent medical professional users most frequently located in Europe or North America (*p* = 0.018). Academic/governmental videos had the shortest time since upload (*p* = 0.028). There were no significant differences with regard to other video characteristics such as views or viewing rate (Table 2).

**Table 2:** Characterization of unique videos by video category

Video category		Academic/ governmental *n* = 63	Independent medical professional users *n* = 9	Other *n* = 13	*p*-value
Host continent, n (%)	Europe	10 (15.9)	1 (11.1)	1 (7.7)	**0.018**
	North America	52 (82.5)	6 (66.7)	8 (61.5)	
	Asia	0 (0.0)	1 (11.1)	2 (15.4)	
	Australia	0 (0.0)	0 (0.0)	1 (7.7)	
	Not given	1 (1.6)	1 (11.1)	1 (7.7)	
Time in months since upload	median [IQR]	22.6 [8.0, 47.1]	47.7 [38.5, 74.4]	44.3 [10.4, 80.3]	**0.028**
Video duration in minutes	median [IQR]	9.0 [4.0, 24.5]	7.0 [5.0, 12.0]	7.0 [4.0, 11.0]	**0.684**
Views	median [IQR]	1411 [452, 9975]	3384 [1002, 5221]	9478 [1635, 16098]	**0.359**
Likes	median [IQR]	23 [10, 125]	84 [37, 110]	124 [15, 236]	**0.278**
Dislikes	median [IQR]	0 [0, 3]	0 [0, 0]	0 [0, 7]	**0.741**
Comments	median [IQR]	1 [0, 10]	6 [2, 33]	3 [1, 32]	**0.231**
Viewing rate	median [IQR]	265.8 [108.7, 569.2]	221.2 [103.1, 293.2]	462.1 [153.4, 1681.4]	**0.554**
Engagement rate	median [IQR]	1.9 [1.2, 3.0]	2.8 [2.0, 3.3]	1.6 [1.2, 2.7]	**0.323**

Data is shown as n (%) or median (IQR, interquartile range). Videos characteristics were compared by video source categories of academic/governmental, independent medical professional users, and other (news/media, industry/for-profit organisation, and non-medical users)

### Video content

Mean content score was 10.5 points (SD 0.6, Table 1). The key features mentioned least frequently were the acute versus chronic course of disease (*n* = 5, 6%), wide geographical variation (*n* = 6, 7%), and need to screen for extrapulmonary disease (*n* = 9, 11%). Information on disease management was often lacking, especially regarding biologics (*n* = 18, 21%) and additional therapies such as rehabilitation (*n* = 20, 24%). Only steroids were named frequently (*n* = 56, 66%). Incorrect or misleading facts were present in 9% of videos (*n* = 8, Table 1). These mainly concerned therapeutic options (e.g. in the sense of steroid therapy always being necessary) and prognosis (e.g. some videos indicated that all patients with sarcoidosis experience pulmonary involvement).

No difference in content between the different video source categories was identified (*p* = 0.824, Table 3). The only trends that emerged were for the key features “unknown aetiology” with best scores for independent medical professional users (*p* = 0.162) and “chronic or progressive disease” with better scores for academic/ governmental videos and other videos (*p* = 0.162).

**Table 3:** Characterization of unique videos by HONCode, DISCERN score and content score

Video source category		Academic/ governmental *n* = 63	Independent medical professional users *n* = 9	Other *n* = 13	*p*-value
HONCode score	Mean (SD)	4.6 (0.9)	3.8 (0.4)	3.9 (0.9)	**0.003**
HONCode, n (%)	Low	0 (0.0)	0 (0.0)	0 (0.0)	**0.551**
	Medium	56 (88.9)	9 (100.0)	12 (92.3)	
	High	7 (11.1)	0 (0.0)	1 (7.7)	
DISCERN score	Mean (SD)	2.3 (0.6)	2.1 (0.5)	2.2 (0.4)	**0.454**
Content score	Mean (SD)	10.2 (5.7)	11.1 (5.9)	11.1 (5.2)	**0.824**

Data is presented as n (%) or mean (SD). HON, Health on the Net; SD, standard deviation

### Video quality

**Fig. 2:**
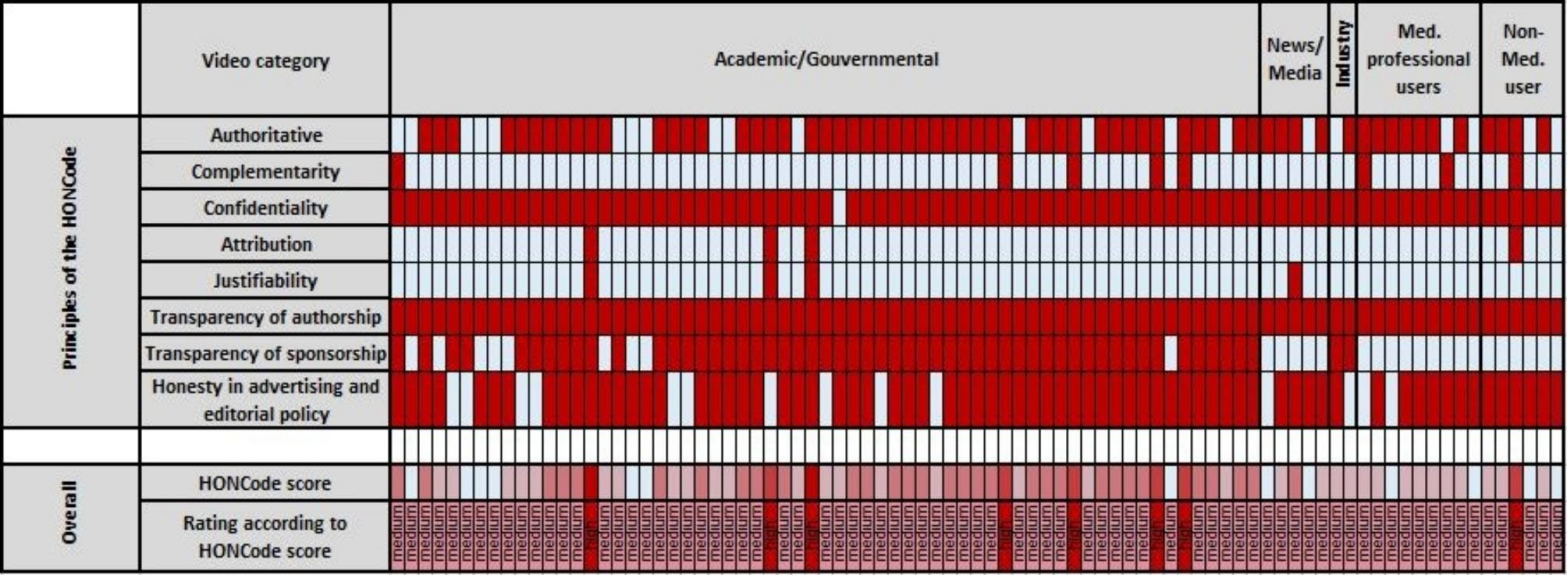
HONCode score by video category. HONCode score items (rows) are presented for single videos (columns, *n* = 85). The HON principle criterium is either met (red) or not met (light blue). HONCode score and HONCode score rating is presented with best scores in dark red and worse scores in light blue. The videos are grouped by video category

General video quality was rated with a mean HONCode score of 4.4 points (SD 0.9, Table 1). Only 9% of the videos demonstrated a high HONCode scoring (*n* = 8), while 91% had a medium HONCode score (*n* = 77, Table 1). HONCode scores in academic/governmental videos were significantly higher than other video source categories (*p* = 0.003, Table 3), especially for the HON category “transparency of sponsorship” (*p* < 0.001). Figure 2 shows HONCode and its items for all 85 videos.

**Fig. 3:**
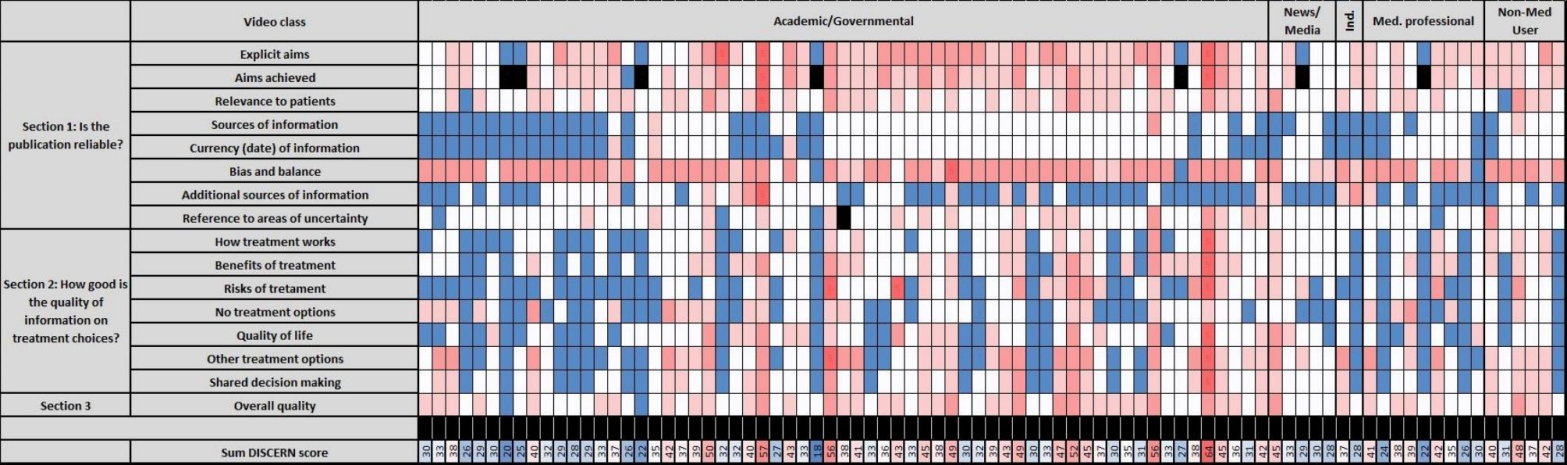
DISCERN score by video category. DISCERN score items (rows) are shown for single videos (columns, *n* = 85). The categorial DISCERN item scoring ranges between 1 (not addressed, blue), 2 (partially adressed, white), 3 (partially addressed, light pink), 4 (partially addressed, dark pink), and 5 (fully addressed, red). The item 2 “aims achieved” was not assessable (NA, black), when item 1 “explicit aims” was scored with 1, i.e. criterion not met. The sum of the DISCERN score ranges between min. 16 and max. 80. The videos are grouped by video category

The median DISCERN score evaluating user-focused video quality was 2.3 points (SD 0.5, Table 1) without significant differences between video categories (*p* = 0.454, Table 3). The DISCERN items “Q1: Are the aims clear?” (*p* = 0.110) and “Q8: Does it refer to areas of uncertainty?” (*p* = 0.167) tended to be better fulfilled in academic/ governmental videos (Fig. 3).

### Association of video ranking and viewing rate with video content and quality

Results of the multivariable linear regression model for the dependent variable video ranking showed an association with the DISCERN score (estimate = 2.5, i.e. increase of the DISCERN score by one point results in an increase of the video ranking by 2–3 points; *p* = 0.012) and the content score (estimate − 4.1, i.e. increase of the content score by one point results in an decrease of the video ranking by 4 points ; *p* = 0.006), but not with HONCode score (*p* = 0.721). However, the model explains only about 10% (models’ multiple R^2^ = 0.107) of the variance of the dependent variable video ranking. There was no association of HONCode score, DISCERN score, and content score with viewing rate (model *p* = 0.194).

On unadjusted analysis, DISCERN score was weak correlated with HONCode score (*r* = 0.35, *p* = 0.001) and moderately correlated with content score (*r* = 0.58, *p* = 0.001). Viewing rate and search rank were negatively correlated (*r*= -0.28, *p* = 0.01).

## Discussion

The internet is a relevant source to obtain health information for patients with sarcoidosis and their caregivers [10]. This study systematically evaluated 85 English-language YouTube videos on sarcoidosis with different validated instruments for content, quality, and reliability. Our aim was to identifythe the main deficiencies of potential YouTube resources on sarcoidosis that future online information about sarcoidosis can seek to address. To our knowledge, this has not been studied to date.

Most analysed videos were from an academic or governmental organisation source, with few videos produced by other source categories. This is in contrast to patient-focused videos in other diseases, such as in IPF, where the source category industry/for-profit organisation was more frequently represented (34% in IPF vs. 2% in sarcoidosis) [11], and similarly in LAM, where non-medical users were more frequently represented (59% in LAM vs. 7% in sarcoidosis) [12]. These differences may be accounted for by industry interest in informing patients about therapeutic options in IPF and by high engagement of patient support groups in LAM. Videos had a median time of 33 months since upload, indicating that some information presented may be out of date, although videos from academic/governmental sources tended to be published more recently. Increased time since video upload may account for the relative lack of information regarding current diagnostic and therapeutic options including biologics as discussed in European Respiratory Society (ERS) 2021 guidelines [5], American Thoracic Society (ATS) guidelines [2] and 2020 Delphi consensus recommendations [16]. Viewing rate and engagement rate achieved values in a medium range. Accordingly, these parameters were higher compared to the very rare disease LAM [12], and even lower in oral candidiasis [19].

Content of YouTube videos on sarcoidosis was poor with a content score of 10.5 out of 25 points. This is particularly evident when compared with a recent analysis of written information on the internet. This study used the same key features on sarcoidosis and the median content score of 19 points was considered to be more acceptable [14]. Here, no differences in content score were found between the different video source categories. Unlike in internet resources for sarcoidosis and YouTube videos for LAM, academic or governmental videos on sarcoidosis did not achieve better content scores [12, 14].

Although accurate and comprehensive delivery of information is particularly challenging in sarcoidosis due to the complexity of the disease and its various organ manifestations [13], most YouTube videos also failed to provide basic information on variable disease course and the need to screen for extrapulmonary disease. This finding supports prior work evaluating information status of patients with sarcoidosis in Germany where authors identified huge information gaps related to the variable natural history of sarcoidosis and disease management [10, 14]. In line with this, another explorative study using semi-structured interviews highlighted information deficits in almost all respondents [20]. In our study, disease management was also inadequately presented in the videos with only 22% and 27% of the videos fully addressing biologics and additional therapeutic options such as rehabilitation respectively. Similar to the analysis of internet resources on sarcoidosis, we found that videos on sarcoidosis only mentioned steroids frequently; however, this was also sometimes misleading in stating that steroid therapy was always necessary [14]. This is similar to findings on YouTube videos in IPF, where 17% of videos presented non-recommended therapies as effective [11]. Together, this work illustrates how health information on YouTube often does not reflect the current state of knowledge and guidelines, and the potential for health information conveyed through this platform to be incorrect or misleading.

In addition to poor content, the quality and reliability of videos regarding sarcoidosis were only moderate. General video quality was rated with a mean HONCode score of 4.4 out of 8 points [17], with 91% of videos was classified as medium quality. HONCode was the only criterion that differed between video source categories with significantly better results seen for academic/ governmental videos. DISCERN score, which focusses on quality assessment of information regarding treatment options [18], had median of 2.3 out of 5 points across 16 domains. This is consistent with the study on internet resources in sarcoidosis also highlighting particularly insufficient quality of information, especially with respect to sources and currency of information as well as information on treatment choices [14]. Strikingly, only four YouTube videos provided sources of information, which might be due to the design and common practice on YouTube [11], but is essential for the reliability of online health information. In the future, newly uploaded YouTube videos on health-specific topics should provide their sources to enable user verification [11]. Our assessments of quality are similar to that seen in other rare diseases including IPF [11] and LAM [12], where quality was found to be inadequate. This is in contrast to YouTube videos on chronic obstructive pulmonary disease (COPD), a common pulmonary disease, where most videos were given high quality rankings [21]. Particularly in health information on rare diseases, there appear to be relevant deficits, so that special efforts are needed here. This could be enabled by state-controlled evaluation of information based on objective criteria. Support from the self-support groups could also be useful as our previous analysis of written online information on sarcoidosis found foundation/ advocacy websites to be best updated [14].

A retrospective study analysed the internet for sarcoidosis-related search terms and found a very high search volume for a rare disease [22]. However, the identification of reliable information by patients poses a particular challenge. This was shown for sarcoidosis both by the above-mentioned German survey [14] and by a Dutch study in which 50% of patients were unable to find adequate information on the disease [23]. These findings are further illustrated by our analyses showing a correlation between video ranking and DISCERN score, but no correlation with HONCode score and even a negative correlation with content. Furthermore, video ranking and viewing rate were negatively associated with each other, meaning that videos with higher search ranks had lower number of views per day since upload. The reasons for these findings are that YouTube ranking is based on views and interaction scores and not on content or quality scores [11]. In contrast, almost two third of sarcoidosis patients trusted the internet as a reliable source of information [10]. This highlights the difficulties in search and evaluation of information on this disease. Patients with sarcoidosis expressed a great wish for education [9] and being involved in treatment decisions as part of shared-decision making [23]. Therefore, they need to have an adequate level of information on their own disease. Studies on cancer highlighted that this level of information is often unavailable on the internet [24].

Our study provides a systematical and broad analysis of YouTube videos based on validated instruments. In particular, content of videos has relevant deficiencies, and overall quality is moderate. Strikingly, the identification of good quality videos is challenging due to the lack of reliable criteria. There were hardly any differences between the video categories in terms of content and quality, and also video ranking and viewing rate failed to identify better videos. To summarise, our analyses highlight that health information on sarcoidosis provided by YouTube videos alone is not sufficient and requires further classification, for example by treating physicians or other sources of information.

However, some limitations applied. Firstly, only English-language videos were considered, and we searched solely the platform YouTube. Therefore, we cannot make any statements about other language information or videos on other platforms. Due to the user-dependant search algorithm used by YouTube, our video rankings may differ from those of other users. We deleted all cookies and the web browsers’ history prior to our initial search to minimize the impact of these factors on search output [25]. Furthermore, our analyses are based on the search term “sarcoidosis”, while possible abbreviations have been disregarded. We developed an expert consensus set of pre-defined key features based on current guidelines for content analysis. In addition, these key features have been used for a previous content analysis of internet resources on sarcoidosis with good results. However, these have not been externally validated. Last, due to lacking demographic data of video consumers, no assessment of the impact of these factors on video content or quality was possible.

## Conclusion

Most YouTube videos present incomplete information of poor quality, particularly missing references and citing specific evidence. Improving patient access to trustworthy and up to date information is needed. Therefore, health information on sarcoidosis provided by YouTube videos requires further classification.

## Electronic supplementary material

Below is the link to the electronic supplementary material.

Supplementary Material 1: **Supplement S1** HONCode score. This instrument is based on eight principles with scoring from 0 for non-fulfilled to 1 for fulfilled [17] [12]. **Supplement S2** DISCERN instrument. Scoring from 1 (quality criterion has not been fulfilled) to 5 (completely fulfilled) [18]. **Supplement S3** List of included videos with video title and search ranks (n= 85).

## Data Availability

No datasets were generated or analysed during the current study.

## Abbreviations

ATS: American Thoracic Society
COPD: Chronic obstructive pulmonary disease
ERS: European Respiratory Society
HDLC: High density lipoprotein cholesterol
HON: Health On the Net
IPF: Idiopathic pulmonary fibrosis
IQR: Interquartile range
LAM: Lymphangioleiomyomatosis
SD: Standard deviation
TC: Total cholesterol
URL: Uniform resource locator
