# Supplementary material for: Is YouTube a sufficient source of information on Sarcoidosis?
Source: Respir Res. 2024 Sep 9;25:334. doi: 10.1186/s12931-024-02956-2 (PMC11386059; doi:10.1186/s12931-024-02956-2)
Supplement: Supplementary file 1 — Supplementary Material 1: Supplement S1 HONCode score. This instrument is based on eight principles with scoring from 0 for non-fulfilled to 1 for fulfilled [17] [12]. Supplement S2 DISCERN instrument. Scoring from 1 (quality criterion has not been fulfilled) to 5 (completely fulfilled) [18]. Supplement S3 List of included videos with video title and search ranks (n= 85). [file 12931_2024_2956_MOESM1_ESM.docx]

**Supplement**

**Supplement S1** HONCode score. This instrument is based on eight principles with scoring from 0 for non-fulfilled to 1 for fulfilled [14] [9].

|  | **Principle** | **Explanation** | **Score** |
| --- | --- | --- | --- |
| **HONCode score** | **1. Authoritative** | Video provides a clear statement about qualifications of the author. | 0-1 |
|  | **2. Complementarity** | Video intends to support, not replace, of the relationship between a patient and existing physician. | 0-1 |
|  | **3. Privacy** | Video preserves confidentiality of data relating to individual patients. | 0-1 |
|  | **4. Attribution** | Video provides clear references to source data and date of last modification. | 0-1 |
|  | **5. Justifiability** | Video discusses the benefits or performance of a specific treatment, and provides appropriate, supporting evidence. | 0-1 |
|  | **6. Transparency** | Video provides contact information for viewers that seek further information or support. | 0-1 |
|  | **Financial disclosure** | Support for this Video is clearly identified. | 0-1 |
|  | **Advertising policy** | Any advertising is clearly identified or labelled. | 0-1 |

**Supplement S2** DISCERN instrument. Scoring from 1 (quality criterion has not been fulfilled) to 5 (completely fulfilled) [15].

| **Section** | **Question** | **Score** |
| --- | --- | --- |
| **Section 1**  **–**  **Reliability** | Q1: Are the aims clear? | 1-5 |
|  | Q2: Does it achieve its aims? | 1-5 |
|  | Q3: Is it relevant? | 1-5 |
|  | Q4: Is it clear what sources of information were used to compile the publication (other than the author or producer)? | 1-5 |
|  | Q5: Is it clear when the information used or reported in the publication was produced? | 1-5 |
|  | Q6: Is it balanced and unbiased? | 1-5 |
|  | Q7: Does it provide details of additional sources of support and information? | 1-5 |
|  | Q8: Does it refer to areas of uncertainty? | 1-5 |
| **Section 2**  **–**  **Treatment choices** | Q9: Does it describe how each treatment works? | 1-5 |
|  | Q10: Does it describe the benefits of each treatment? | 1-5 |
|  | Q11: Does it describe the risks of each treatment? | 1-5 |
|  | Q12: Does it describe what would happen if no treatment is used? | 1-5 |
|  | Q13: Does it describe how the treatment choices affect overall quality of life? | 1-5 |
|  | Q14: Is it clear that there may be more than one possible treatment choice? | 1-5 |
|  | Q15: Does it provide support for shared decision-making? | 1-5 |
| **Section 3**  **–**  **overall rating** | Q16: Based on the answers to all of the above questions, rate the overall quality of the publication as a source of information about treatment choices. | 1-5 |
| **DISCERN score** | | **16-80** |

**Supplement S3** List of included videos with video title and search ranks (n= 85).

| **Video titel** | **Website URL** | **Search rank** |
| --- | --- | --- |
| Sarcoidosis - causes, symptoms, diagnosis, treatment, pathology | https://www.youtube.com/watch?v=9p3UVhk3TWU | 1 |
| Understanding Sarcoidosis: A Visual Guide for Students | https://www.youtube.com/watch?v=p3D7MHr8dr0 | 2 |
| Sarcoidosis, Causes, Signs and Symptoms, Diagnosis and Treatment. | https://www.youtube.com/watch?v=KFJ9j8cJmX4 | 3 |
| Sarcoidosis \| Pathophysiology, Diagnosis, Treatment | https://www.youtube.com/watch?v=JPG2GwLMHUs | 4 |
| What is Sarcoidosis? | https://www.youtube.com/watch?v=-yLrBPD2HV8 | 5 |
| Sarcoidosis \| Restrictive Lung Disease \| Pulmonology | https://www.youtube.com/watch?v=m9rcVK0J8GI | 6 |
| Understanding Sarcoidosis and How It Affects People | https://www.youtube.com/watch?v=oc_LZunuUE0 | 9 |
| Sarcoidosis Patient Story | https://www.youtube.com/watch?v=6U7OpgFojaw | 10 |
| Sarcoidosis - Causes, Symptoms and Treatment | https://www.youtube.com/watch?v=bRZlBUkiqsE | 11 |
| Sarcoidosis | https://www.youtube.com/watch?v=0QAdMxyNekk | 12 |
| Sarcoidosis Diagnosis and Management \| Restrictive Lung Disease \| Pulmonology | https://www.youtube.com/watch?v=x3CaGqTrLrg | 13 |
| Sarcoidosis -- a Mystery Disease? | https://www.youtube.com/watch?v=WOG7QrZIfGk | 15 |
| Sarcoidosis 101 \| American Lung Association | https://www.youtube.com/watch?v=9VF0hud9rW8 | 16 |
| Living with Sarcoidosis | https://www.youtube.com/watch?v=tNxCKQersNA | 19 |
| Behind the Mystery of Sarcoidosis: A Rare Disease That Can Cause Shortness of Breath, Chest Pain | https://www.youtube.com/watch?v=u80fX8dPLHY | 20 |
| Diagnosis to Management: What You Need to Know about Living with Sarcoidosis | https://www.youtube.com/watch?v=0U_k__mQeP8 | 23 |
| Sarcoidosis in Neuro-Ophthalmology | https://www.youtube.com/watch?v=hGsFS9Eg8uk | 28 |
| John Carlin opens up about his battle with sarcoidosis | https://www.youtube.com/watch?v=ThOLWnxszSE | 30 |
| American Lung Association - Sarcoidosis | https://www.youtube.com/watch?v=yEN21v_I9EA | 31 |
| SPD 2022: Session 2 – Advanced Pulmonary Sarcoidosis | https://www.youtube.com/watch?v=Vu7EzWdqBxg | 32 |
| Cardiac Sarcoidosis Symptoms | https://www.youtube.com/watch?v=NGfWLmppEsQ | 33 |
| Living with and Managing Sarcoidosis | https://www.youtube.com/watch?v=_116EyP1XKA | 34 |
| Sarcoidosis and Heart Rhythm Testing | https://www.youtube.com/watch?v=ezHBhfQzqRg | 35 |
| Sarcoidosis Questions Answered | https://www.youtube.com/watch?v=7W1L4HBPdvw | 36 |
| Sarcoidosis: But You Don't Look Sick | https://www.youtube.com/watch?v=GG0YP2F3S-E | 39 |
| Education Webinar Series: Sarcoidosis of the Spine | https://www.youtube.com/watch?v=zKjmIwJL42o | 40 |
| Cardiac Sarcoidosis \| Part 2: Diagnosis and Treatment | https://www.youtube.com/watch?v=L6BlBgzJldk | 41 |
| Pulmonary Sarcoidosis: Diagnosis and Treatment | https://www.youtube.com/watch?v=CzRDT-Kim3I | 42 |
| Overview of Pulmonary Sarcoidosis | https://www.youtube.com/watch?v=h3k8N-AbqzY | 43 |
| Sarcoidosis Stories: Rick's Story | https://www.youtube.com/watch?v=NtXUPLucIhM | 45 |
| Sarcoidosis easy explained; causes, mechanism and pathology | https://www.youtube.com/watch?v=MxUne3vHxQk | 47 |
| Cardiac Sarcoidosis \| Dr Thillai | https://www.youtube.com/watch?v=0oaJhRh9UXk | 50 |
| What is cardiac sarcoidosis? | https://www.youtube.com/watch?v=yIF2hw4nQqA | 52 |
| Steroids for Treating Sarcoidosis an Option but Far From Ideal | https://www.youtube.com/watch?v=_O7cCQw3u9A | 53 |
| SPD 2022: Session 3 - Cardiac Sarcoidosis | https://www.youtube.com/watch?v=Ukg_Tk10eb8 | 54 |
| Sarcoidosis - An Overview | https://www.youtube.com/watch?v=JOHQgmqpNY8 | 55 |
| FAQs- Sarcoidosis and COVID-19 | https://www.youtube.com/watch?v=y0A69EvzrpQ | 56 |
| Cutaneous Sarcoidosis | https://www.youtube.com/watch?v=CnALWzf7O00 | 58 |
| Sarcoidosis - 10 Tips to Identify and Treat | https://www.youtube.com/watch?v=e53ahFnMCc8 | 62 |
| Sarcoidosis | https://www.youtube.com/watch?v=NuQTUnitJus | 64 |
| Vitamin D and Sarcoidosis: What you need to know - Sarcoid Tip with Dr. Harris | https://www.youtube.com/watch?v=w6SmbY7WUi4 | 67 |
| Implantable Devices for Patients with Cardiac Sarcoidosis | https://www.youtube.com/watch?v=QqUSYnRxLxo | 68 |
| Pulmonary Sarcoidosis Q&A \| Temple Lung Center | https://www.youtube.com/watch?v=O4mqLvAyNhM | 75 |
| Town Hall: Sarcoidosis and Steroids | https://www.youtube.com/watch?v=dNwbcBIyQhE | 78 |
| What is Sarcoidosis? | https://www.youtube.com/watch?v=1iU0xvrNBQQ | 79 |
| Complex and Progressive Sarcoidosis \| Prof Wells (Part 2) | https://www.youtube.com/watch?v=5_N5XhxZy5w | 87 |
| How the Diagnosis of Sarcoidosis is Made - Dr Elisabeth Carr | https://www.youtube.com/watch?v=dT-cP5-CsPs | 88 |
| Q&A Session – Neurosarcoidosis Patient Day 2022 | https://www.youtube.com/watch?v=VwPObOvBBHg | 89 |
| Understanding the latest sarcoidosis treatment recommendations: Patient webinar | https://www.youtube.com/watch?v=75l9E5qa6eg | 92 |
| Stages of Sarcoidosis with Dr. Carr | https://www.youtube.com/watch?v=XfOzthQxaaY | 94 |
| Remission in Sarcoidosis with Dr. Carr | https://www.youtube.com/watch?v=GWMFkSbYgVQ | 98 |
| Dr. Deborah Parks- Muskuloskeletal Sarcoidosis | https://www.youtube.com/watch?v=jkJl9Y-dzLI | 101 |
| Neurosarcoidosis \| Dr Kidd (Part 1) | https://www.youtube.com/watch?v=MtvjgPw9peQ | 103 |
| SARCOIDOSIS | https://www.youtube.com/watch?v=FPqwwsHTlHE | 104 |
| Sarcoidosis and the Eye hosted by Dr. Meghan Berkenstock | https://www.youtube.com/watch?v=UDpNX3bNFLc | 105 |
| A Holistic Approach To Sarcoidosis | https://www.youtube.com/watch?v=mpLti-Sj5d8 | 111 |
| Sarcoidosis Overview \| American Lung Association | https://www.youtube.com/watch?v=xZPtfoHeO3s | 113 |
| Covid-19/Sarcoidosis Q&A with Dr Robina Coker and Henry Shelford. | https://www.youtube.com/watch?v=377zBPn55uA | 117 |
| Dr. Lauren Graham - Cutaneous Sarcoidosis | https://www.youtube.com/watch?v=MxIoMXykfUU | 118 |
| To Be Sarcoidosis | https://www.youtube.com/watch?v=GjruFgMWB9k | 119 |
| Dr. Bob Baughman - Sarcoidosis and Prednisone | https://www.youtube.com/watch?v=TxL1PxxjrCo | 121 |
| Sarcoidosis \| Pathology \| Immunology \| Med Vids made simple | https://www.youtube.com/watch?v=Pb4Wa3B-VoA | 122 |
| What are the Longterm Effects of Sarcoidosis Medications with Dr. Raj: Sarcoid Tip | https://www.youtube.com/watch?v=yO_mX4lFnI0 | 125 |
| Cardiac MRI for Patients with Cardiac Sarcoidosis | https://www.youtube.com/watch?v=fyDCZol-XbA | 129 |
| Lanier O’Hare - Sarcoidosis-related Fatigue and Depression | https://www.youtube.com/watch?v=hzBNimRt2gQ | 131 |
| Cleveland Conference 2018: Advanced Sarcoidosis - Daniel Culver | https://www.youtube.com/watch?v=fC--8R5TRQk | 133 |
| Is my Sarcoidosis Treatment is Working with Adam Morgenthau M D | https://www.youtube.com/watch?v=ej6Cl19poLs | 137 |
| Stomach Issues Related to Sarcoidosis? with Dr Raj Dasgupta: Sarcoid Tips | https://www.youtube.com/watch?v=0aNxBJDvG6A | 138 |
| How is Sarcoidosis treated (include different types of medicines)? | https://www.youtube.com/watch?v=CE56kYjFdFs | 141 |
| Cardiac Sarcoidosis Patient Day Session 2 | https://www.youtube.com/watch?v=TREJnGy-DFM | 145 |
| What you need to know about Sarcoidosis & the kidneys | https://www.youtube.com/watch?v=lSM9uzTbw2w | 162 |
| Sarcoidosis, pathophysiology clinical aspects and management | https://www.youtube.com/watch?v=D-ahTqbqnqE | 163 |
| Is there a Cure for Sarcoidosis with Dr Raj Dasgupta: Sarcoid Tips | https://www.youtube.com/watch?v=wGYaHm3fIR0 | 167 |
| The Sarcoidosis Vlogs #1 - I Have Sarcoidosis - Level 4 Pulmonary Sarcoidosis | https://www.youtube.com/watch?v=4yiNHPNeRZs | 168 |
| Treating Arthritis in Sarcoidosis with Dr. Raj: Sarcoid Tips | https://www.youtube.com/watch?v=x-NK31keNHk | 170 |
| The Royal Brompton and SarcoidosisUK Sarcoidosis Patient Day - YouTube | https://www.youtube.com/watch?v=25yqKmwm5Fo | 178 |
| Sarcoidosis I Medicine I For NEET-PG 2021 AIIMS I By Dr. Shadab Moosa, National Level Faculty | https://www.youtube.com/watch?v=5jh_kNwIOW4 | 182 |
| Sarcoidosis Part 1 | https://www.youtube.com/watch?v=7ynFkbWgZE0 | 183 |
| Sarcoidosis Treatments- Leslie Cooper | https://www.youtube.com/watch?v=yt5YFqHr8Ts | 184 |
| What is the effect of Sarcoidosis on lymph glands? | https://www.youtube.com/watch?v=-mB8BA1S2dA | 189 |
| Cleveland Conference 2018: Neurosarcoidosis - Dr. Mary Willis | https://www.youtube.com/watch?v=Kpvqs0-9Gms | 194 |
| Sarcoidosis–What are my Treatment Options? | https://www.youtube.com/watch?v=oz2iSXv6Pkc | 196 |
| Session 4 - The Royal Brompton and SarcoidosisUK Sarcoidosis Patient Day | https://www.youtube.com/watch?v=_5E1A31vSIM | 198 |
| Black & Rare: Sarcoidosis in Black Women - YouTube | https://www.youtube.com/watch?v=T-G1D2ONnys | 199 |
| What In the World is Sarcoidosis and Why Can't I Breathe? | https://www.youtube.com/watch?v=OT1cyiw6EBE | 200 |
